# Brain‐Inspired Polymer Dendrite Networks for Morphology‐Dependent Computing Hardware

**DOI:** 10.1002/advs.202502291

**Published:** 2025-08-11

**Authors:** Corentin Scholaert, Yannick Coffinier, Sébastien Pecqueur, Fabien Alibart

**Affiliations:** ^1^ IEMN UMR 8520 Univ. Lille CNRS Univ. Polytechnique Hauts‐de‐France Lille 59000 France; ^2^ Laboratoire Nanotechnologies & Nanosystèmes (LN2) CNRS IRL‐3463, 3IT Sherbrooke Québec J1K0A5 Canada

**Keywords:** dendritic networks, electropolymerization, morphology‐dependent hardware, unconventional computing

## Abstract

Process variation is always a challenge to mitigate in electronics. This especially holds true for organic semiconductors, where reproducibility concerns hinder industrialization. Challenging this concept, it shows AC‐electropolymerization to be a powerful platform for the development of morphology‐dependent computing hardware, thanks precisely to its intrinsic stochasticity. The findings reveal that electropolymerized polymer dendrite networks exhibit a complex structure‐operation relationship that allows to implement nearly linear to nonlinear functions. Moreover, dendritic networks can integrate a limitless number of inputs from their environment, which can be used to the advantage in the context of in materio computing to discriminate between different spatiotemporal inputs. These results position electropolymerization as a pivotal technique for the bottom‐up implementation of computationally powerful objects. This study anticipates this study to help shifting the negative perception of variability in the material science community and promote the electropolymerization framework as a foundation for the development of a new generation of hardware defined by its topological richness.

## Graphical abstract

Although process variability is often perceived as a drawback in electronics, this work harnesses the stochastic nature of electropolymerization as a powerful ally for computation. The resulting conductive polymer dendrites exhibit unique structure‐property relationships and support *in memory* computing, paving the way for the development of a new class of evolving, morphology‐dependent wetware.

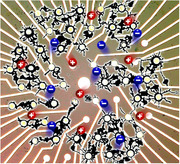

## 1 Introduction

Silicon‐based digital computing technologies have achieved tremendous levels of sophistication and perfection over a relatively short period of time in the realm of digital information processing. Yet, digital computing is only a small corner of the much larger field of general computing. For some time now, researchers have proposed unconventional approaches to computing, redefining the notion as the establishment of input‐output relationships and leveraging the intrinsic physics of virtually any physical system to carry out computing tasks.^[^ ^1^, ^2^, ^3^, ^4^, ^5^ ^]^ Within the general framework of unconventional computing, brain‐inspired approaches have lately started to attract a lot of attention.^[^ ^6^, ^7^, ^8^, ^9^, ^10^ ^]^ Neuromorphic computing could offer some answers to the challenges faced by digital computing as the brain is able to massively parallelize information processing at a very low energy cost, all the while being robust and adaptable thanks to its structural and functional plasticity.

Recently, a new generation of neuromorphic devices based on organic electronic materials has been developed, drawing inspiration from and mimicking the operation of the brain.^[^ ^11^, ^12^, ^13^, ^14^, ^15^, ^16^, ^17^, ^18^, ^19^ ^]^ These devices hold the potential to reach ultralow energy consumption,^[^ ^20^, ^21^ ^]^ and are easier to process than traditional silicon‐based technology.^[^ ^22^ ^]^ When it comes to fabrication, one of the most interesting aspects of organic electronics is that monomers can simply be polymerized, which renders the fabrication process much easier. Over the past few years, electropolymerization has proven an increasingly promising fabrication technique, allowing for a more adaptative bottom‐up approach. It opens the way for the easy and versatile deposition of conductive polymer coatings,^[^ ^23^, ^24^, ^25^, ^26^, ^27^ ^]^ which have been used to implement various neuromorphic features, such as sensory devices,^[^ ^28^, ^29^ ^]^ plastic synapses^[^ ^15^, ^19^ ^]^ and spiking neurons,^[^ ^30^ ^]^ effectively demonstrating the robustness of such materials for computing applications. Yet, in these cases, organic electrochemistry is only perceived as an alternative tool for the deposition of polymer thin films.

One of the main drawbacks of organic electronics has always been process variation, in part due to the phase changes associated with the low glass transition temperature of soft matter. While this inherent variability may pose a challenge for sensing applications, it could however prove advantageous for computing purposes. Indeed, electropolymerization on organic electrochemical transistors (OECTs) has been exploited to generate structurally different active materials on lithographically patterned devices in order to enrich the dynamics of a reservoir, thus facilitating classification tasks.^[^ ^31^ ^]^ Because each OECT of the array was unique, information was projected on a wider dimensional space. More recently, networks of electropolymerized polymer fibers have been shown to implement Hebbian learning^[^ ^32^ ^]^ and Boolean logic,^[^ ^33^ ^]^ as well as biosignal classification through the reservoir computing framework.^[^ ^34^ ^]^ These systems appear to be interesting candidates for the implementation of unconventional computing frameworks, capitalizing on the complex dynamics at work within these networks of artificial dendrites. However, to this day, organic devices still do not exploit the topological diversity offered by the electropolymerization framework. The rich structural variety offered by this bottom‐up approach could be taken advantage of to perform *in materio* computing based on the unique behavior of each device. Therefore, electropolymerization is not only a cost‐effective alternative to otherwise expensive fabrication techniques, but also a whole new avenue for the conception of a class of a neuromorphic hardware with morphology‐dependent functions.

In this article, we explore the possibilities offered by poly(3,4‐ethylenedioxythiophene):polystyrene sulfonate (PEDOT:PSS) fiber networks from an unconventional computing point of view. We characterize and exploit the topological richness of electrogenerated conducting polymer dendrites for *in liquido* computation. In particular, electrochemical self‐gating and inter‐gating effects cause voltage‐to‐current nonlinear transformations that can be used to our advantage to process information. We show that these systems exhibit an inherent form of memory that can be electrochemically programmed by specifically organizing the ionic distribution in the polymer network. Finally, we show that such networks function as electrochemical classifiers, where spatiotemporal projections of nonlinearly separable input patterns can be discriminated thanks to the higher dimensional projection inherent to the dendritic network complexity. These properties make such dendritic networks advantageous for the development of hardware for artificial intelligence such as the physical implementation of reservoirs, and are also sought‐after elements for the realization of Physical Unclonable Functions (PUF) with embedded computational functionalities.^[^ ^35^ ^]^

## 2 Results

### 2.1 Nonlinear Behavior of Physically Connected Dendrites

Previous studies had brought to light that conducting polymer dendrites behave like OECTs: applying a positive voltage to the gate electrode of the system leads cations into the bulk of the material, thus dedoping the PEDOT:PSS channel.^[^ ^36^ ^]^ So far, these studies were mostly limited to one electrochemical transistor, with a continuous dendritic channel grown between two electrodes. Cucchi and coworkers went a step further and took advantage of the nonlinearities observed in networks of polymer fibers to implement reservoir computing.^[^ ^34^ ^]^ In their implementation, the authors took advantage of feedback connections to increase the nonlinearity of the spatiotemporal signal projection, thus requiring additional circuitry overhead. In this article, we present a solution in which nonlinearities are induced solely by the complexity of the network topologies, giving rise to self‐gating and inter‐gating effects mediated through the common electrolyte.

**Figure 1:**
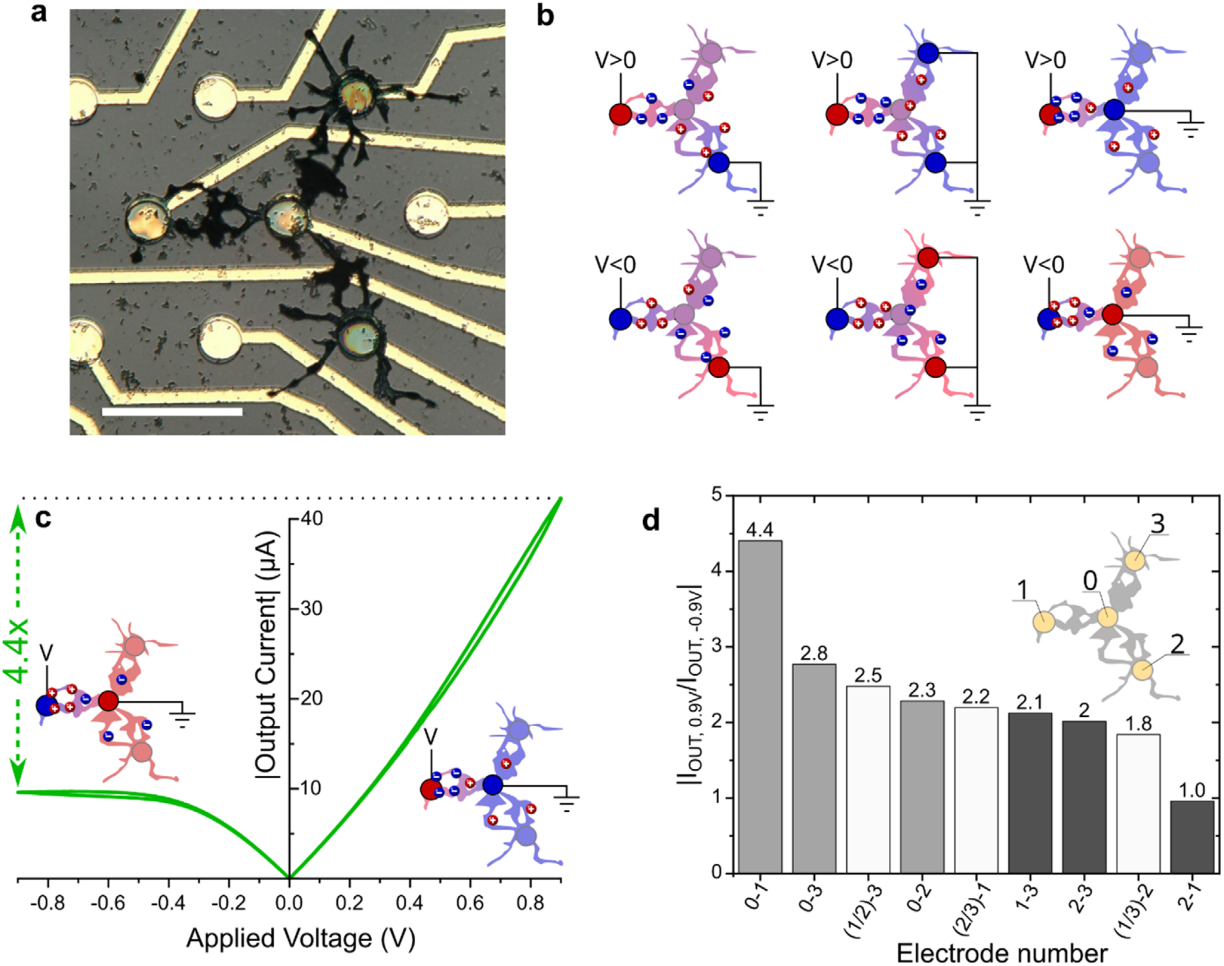
Nonlinear behavior and self‐gating effect of a multiterminal dendritic OECT. a) Microscopic photo of a four‐terminal dendritic system. Scale bar = 100 µm. b) Schematic of the ionic distribution within the system when different potentials are applied. Relative potential is represented by the color scale, red being the point of highest potential in the system while blue is the lowest one. c) Output characteristic of the Y‐shaped device pictured in (a). The curve shows an asymmetric electrical behavior related to the ionic distribution in the system, as illustrated in the subset figures. When the applied voltage is negative, the output current reaches a plateau at ≈0.4 V, whereas it remains linear when V > 0 and up to 0.9 V. The rectification coefficient, computed as |*I_OUT_* |_0.9*V*_/|*I_OUT_* |_−0.9*V*_, is equal to 4.4. d) Breakdown of the asymmetry ratio as a function of the polarized terminals. The x‐axis indicates which electrodes are addressed in each configuration (e.g., (1/2)‐3). The grounded electrode is always written to the left of the hyphen (electrodes 1 and 2 are shorted), and the terminal undergoing voltage sweep is always written to the right (electrode 3). The numbers correspond to the electrodes indicated in the inset of the figure. The wiring corresponding to this nomenclature can be found in Figure S2 (Supporting Information).

Within a network of physically connected polymer dendrites immersed in an aqueous electrolyte, the distribution of potentials plays a key role, altering the behavior of the system through cross‐talking between the different fibers of the network. **Figure** **1** presents the nonlinear electrical behavior of a Y‐shaped dendritic system grown between four gold electrodes of a typical MultiElectrode Array (MEA), operated within a water‐based electrolyte (Phosphate Buffered Saline, PBS). Figure 1b depicts how the behavior of the system can be influenced through voltage distribution across the system by carefully choosing which electrodes to address. It is important to note that the gold lines that can be observed in Figure 1a are covered by a thick layer of insulating material (see Experimental Section/Methods for more details regarding the fabrication process, as well as Figure S1 (Supporting Information) for a cross‐sectional diagram of the device), preventing any coupling with the parts of the system that are in contact with the electrolyte. Depending on the polarization of the nodes of the circuit, the electrochemical doping profile (supported by the cationic charges in water, mostly sodium cations in the present case) within the different PEDOT:PSS fibers can be modified. This allows the control of different conductance profiles within a single dendritic network inducing various transport paths in the material, which is made possible only because the object has a shape of its own. As such, a polymer dendrite cannot be considered as a discrete element with a well‐defined number of inputs and outputs, since these devices exhibit a direct structure‐property relationship.

Figure 1c shows the asymmetric behavior of the Y‐shaped network when pinning the central electrode of the dendritic system to the ground while varying a single input between −0.9 and 0.9 V. When the input voltage was negative, cations closed the channel between the input and the dendrite core. As a single input channel has a residual volume compared to the full dendritic system, the total capacitance of the dendrites that did not participate in the conduction of the current was superior to that of the channel separating the single input from the central electrode of the system. This led to a self‐gating effect on a single dendritic extension, with cations accumulating in the conduction path, leading to a plateau in the current at ≈‐0.4 V. On the other hand, when the input potential V was positive, thanks to the distribution of capacitance within the system, the cations were shared among the three dendrites of the multiterminal OECT. Consequently, the dedoping of the conduction dendrite was far less important than observed when V < 0 V, and its conductance remained stable up to 0.9 V. In addition, a clear hysteresis phenomenon can be observed, exhibiting a memory effect associated with the ionic dynamics in the system. Although reminiscent of the behavior of memristors,^[^ ^37^ ^]^ this memory was in this case volatile. Yet, this observation holds potential for the integration of such multiterminal systems. Conventional memristors are not self‐rectifying and are typically coupled with transistors to ensure isolation and avoid leakage in memory cells.^[^ ^38^ ^]^ Although this study did not explore this direction, investigating how these self‐rectifying behaviors emerge in multiterminal components and how to exploit them could be highly valuable in future research.

This Y‐shaped system thus can be biased with an applied voltage in a forward or a reverse polarization. The asymmetry coefficient, defined here as |*I_OUT_* |_0.9*V*_/|*I_OUT_* |_−0.9*V*_, is presented for all the permutations of the applied potential and ground in the system. Figure 1d presents the different ratios obtained in these different configurations. The highest coefficient was obtained for the previously discussed case, with a ratio of 4.4. On the other hand, when the lower right electrode was grounded and V was applied to the left electrode, a symmetrical (although nonlinear) behavior was observed, resulting in a ratio of one (all the plots for the different cases can be found in Figure S2, Supporting Information). These differences further highlight the intricate relationship between topology and nonlinearity: the specific location of the input conditions the output of the system.

### 2.2 Inter‐Gating Effect

If, as seen previously, several dendrites physically sharing the same node are intricated, their electrochemical coupling with the common electrolyte is also a frequent challenge as it induces cross‐talking.^[^ ^39^ ^]^ Because of the common electrolyte, one of the most critical technological challenges for OECTs is the ability to independently address each device of the matrix, which is required to retrieve information from a single element. This issue can be addressed at the expense of more complex fabrication procedures.^[^ ^40^, ^41^ ^]^

**Figure 2:**
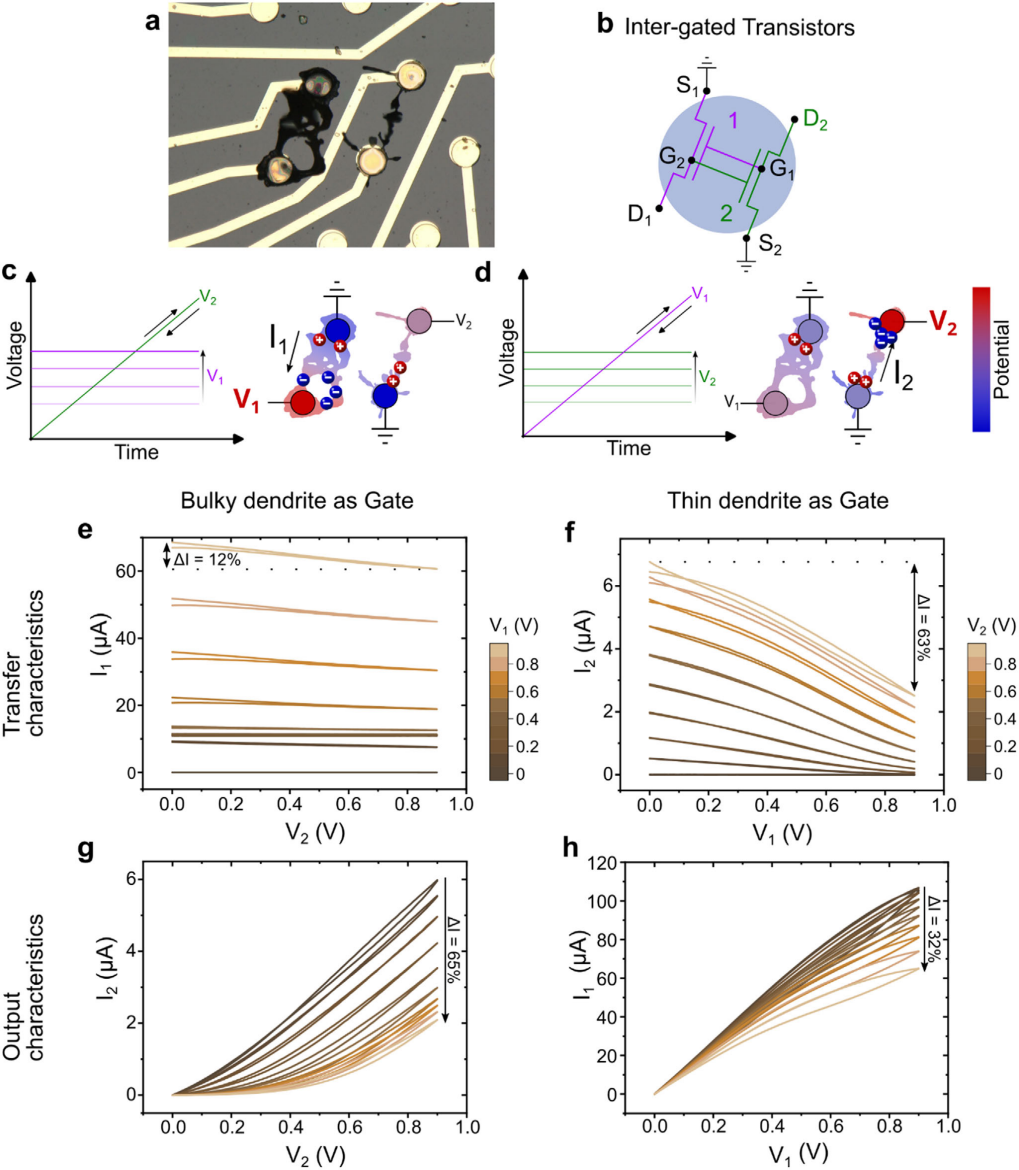
Inter‐gating effect between two parallel dendritic OECTs. The morphology of the PEDOT:PSS fibers is of primary importance. a) Microscopic photo of the two dendrites used as OECTs, showing that the one on the left (grown at 25 Hz) is noticeably thicker than the one on the right (grown at 200 Hz). b) Equivalent circuit of the two parallel transistors. The PEDOT:PSS channel of each OECT acts as the gate for the other transistor. c,d) Ionic distribution when the bulky and thin dendrites are respectively used as the gate. The color scale indicates the nodes of highest potential (red) and lowest potential (blue) in the circuit. e,f) Transfer characteristics of the thin and bulky dendrites, respectively. g,h) Output characteristics of the thin and bulky dendrites, respectively.

On the other hand, cross‐talking between devices can also be apprehended as a means of communication between physically disconnected parts of the system. As PEDOT:PSS is a good material for efficient OECT gating,^[^ ^42^, ^43^ ^]^ two dendritic transistors operating simultaneously should exert a gating effect on each other, as displayed in **Figure** **2**.

The influence of operating a dendritic device in the presence of neighboring fibers was assessed by growing two parallel dendrites, with the first being noticeably thicker than the second (Figure 2a).

The dendrites were operated simultaneously and characterized as OECTs, with their transfer and output characteristics recorded (Figure 2e–h). Once again, the morphology of the dendrites appears to play a major role in the way the whole system behaves. Indeed, using the thicker dendrite as the gate rather than the thinner one (V_1_ fixed while V_2_ was swept, see Figure 2c) had a greater impact on the output current. Figure 2g shows that as V_1_ increases, the drain current I_2_ gradually decreases. The same trend was also observed when the roles were reversed and the thinner dendrite was used as the gate (see Figure 2h), although to a lesser degree: in the former case, the drain current decreases by 65% when V_1_ = 0.9 V versus when V_1_ = 0 V, compared to ≈32% in the latter case (Figure 2h).

The shape of the output characteristics is also distinctly different. When the bulky dendrite was used as the gate, I_2_ remained very low up to ≈0.4 V, before entering a linear region. Interestingly, this was not observed in the second case (Figure 2h), during which the output current followed a linear trend before starting to saturate, although the saturation region was not reached yet at 0.9 V. Once again, the morphology of the dendrites and their ability to retain ions might be at the origin of this phenomenon. With its large volumetric capacitance, PEDOT:PSS is an effective gate material that can efficiently promote doping/dedoping. The voluminous fiber could thereby dedope its neighboring dendrite almost entirely at a high enough V_1_, as the lowest potential of the system would in that case be the grounded electrode of the thin dendrite (Figure 2c). V_2_ had to overcome a certain threshold to be able to move ions back into the electrolyte and to start regenerating the initial high doping state of the dendrite. When the roles were reversed (Figure 2d), the thin dendrite also played the role of the gate of the system, although less efficiently than its more voluminous counterpart as it was not able to close entirely the channel of the second transistor within this voltage range.

So far, the previous sections have established that a network of conductive polymer dendrites behaves as a multiterminal transistor, within which the dendrites are not a collection of independent elementary nodes, but are rather interconnected and interdependent objects. A system made of multiple physically connected dendrites experiences a self‐gating effect, while two independent devices sharing the same electrolyte also undergo a similar inter‐gating effect. As a consequence of this cross‐talking, computing can be implemented on systems of electrically interconnected dendrites (forming a single continuous topology) as well as systems of distinctive dendritic topologies electrochemically coupled through an electrolyte.

### 2.3 Multiply‐Accumulate Operation with Multiterminal OECTs

**Figure 3:**
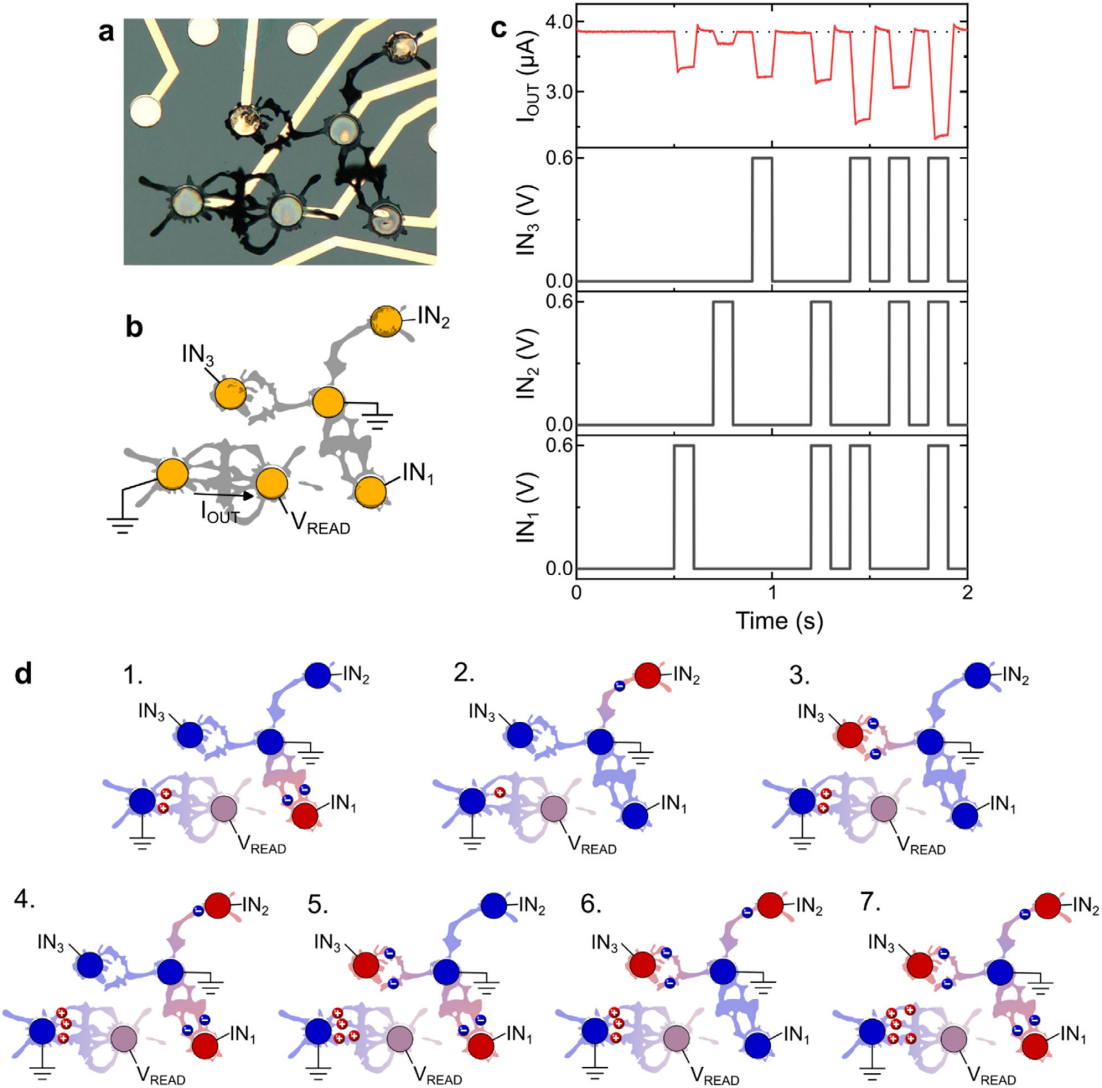
Real‐time computing, taking advantage of the nonlinearities and inter‐gating effect of dendritic OECTs. a) Microscopic photo and b) schematics of the system showing the three inputs and the output. The Y‐shaped device is used as the input, while the output current is read at the drain of the single dendrite (V_READ_ = 100 mV). c) Output current and input voltages as a function of time. Transients were manually removed to focus on the steady‐state response of the circuit. d) Ionic distribution schematizing the dedoping mechanisms in the system when the inputs are polarized. The color scale indicates the nodes of highest potential (red) and lowest potential (blue) in the circuit.

Taking advantage of the inherent properties of dendritic networks, a multiterminal system was used to implement the multiply‐accumulate (MAC) function (**Figure** **3**). Monitoring only one output, this dendritic network allowed determining the combination of inputs that was activated. The circuit was made of a Y‐shaped device, and the output current was read from the drain electrode of a single dendrite (Figure 3a,b), all immersed in PBS. Pulses of voltage (0.6 V, 200 ms) were sent as the inputs to the outermost electrodes of the Y‐shaped device, the central terminal being grounded, in the fashion depicted in Figure 3c. A small potential was continuously applied to the output electrode to monitor the variations of the output current (V_READ_ = 100 mV).

As a result of the positive voltage pulses, the output dendrite experienced electrochemical dedoping due to the movement of ions through the electrolyte, as schematized in Figure 3d. The modulation of current was characteristic of the activated input node: the output current underwent a 13% modulation for IN_1_, 5% for IN_2_ and 18% for IN_3_. The magnitude of the modulation appears to be related to the morphology of the dendrites, as seen in Figure 2, as well as the distance that separates the input node from the output dendrite, the third input being the closest one to the output dendrite while IN_2_ was the farthest away. This dependence of the gating effect with the distance from the drain electrode has already been observed in thin‐film OECTs, and has proven useful for orientation selectivity.^[^ ^44^ ^]^ Yet, in the present system, it appears impossible to disentangle the influence of the fiber morphology from that of the distance.

As seen in Figure 3c, every single combination of inputs had a unique signature that could be sensed by the output dendrite. The more inputs were activated, the more important was the modulation of the output current. Indeed, when all three inputs were set to 0.6 V, this modulation reached a maximum of 38%. With a readout from a single node in the circuit, the system was able to discriminate activated inputs in a quasi‐linear fashion. Such operation reproduces the multiply‐accumulate (MAC) function that is a key operation in artificial neural networks. In this particular implementation, the synaptic weights were defined by the topology of the dendrites belonging to the Y‐shaped device, while the accumulation was implemented with the readout device. Nevertheless, it is important to note that the randomness of dendritic growth implies that precisely controlling the weight of each dendrite is not straightforward. As it has been demonstrated, the morphology of dendritic objects can be controlled globally. However, at the local scale, the stochastic nature of electropolymerization intrinsically promotes large variabilities, conflicting with the idea of physical reproducibility (and *a fortiori* of the reproducibility of electrical properties). Moreover, when operating with a higher number of polarized nodes in the system, growing a perfectly predefined network of dendritic OECTs can become delicate. From a general perspective, the MAC operation corresponds to a linear function that could be rationalized for a small topological dendritic system (i.e., three branches of the Y‐shaped device). In the following of the discussion, we show that more complex topologies can lead to nonlinear functions that increase the computational power of the dendritic networks.

### 2.4 *In Materio* Computing

The influence of the system on itself and on the nearby PEDOT:PSS dendrites, as well as its ability to retain information over time and discriminate past voltage events, are essential ingredients for the realization of complex information processing tasks. Indeed, performing time series prediction thanks to the nonlinear dynamics of such an organic electrochemical network,^[^ ^34^ ^]^ coupled with the structural plasticity offered by this type of hardware,^[^ ^45^ ^]^ makes dendritic networks an exciting candidate to instill a new direction in the search of neuromorphic material. In this sense, conductive polymer networks could find themselves at the crossroad between different domains of research, borrowing ideas both from the biological realm (neurogenesis and synaptic plasticity) and computing. Yet so far, if dendritic networks appear to be a very useful tool to project information in a reservoir of higher dimension, a subsequent layer of machine learning is still required to perform classification.^[^ ^34^ ^]^ In this work, we propose a new framework based solely on hardware, capable of *in memory* spatiotemporal information processing, with the output condensed into a single value of current.

#### 2.4.1 Spatial Information Processing

**Figure 4:**
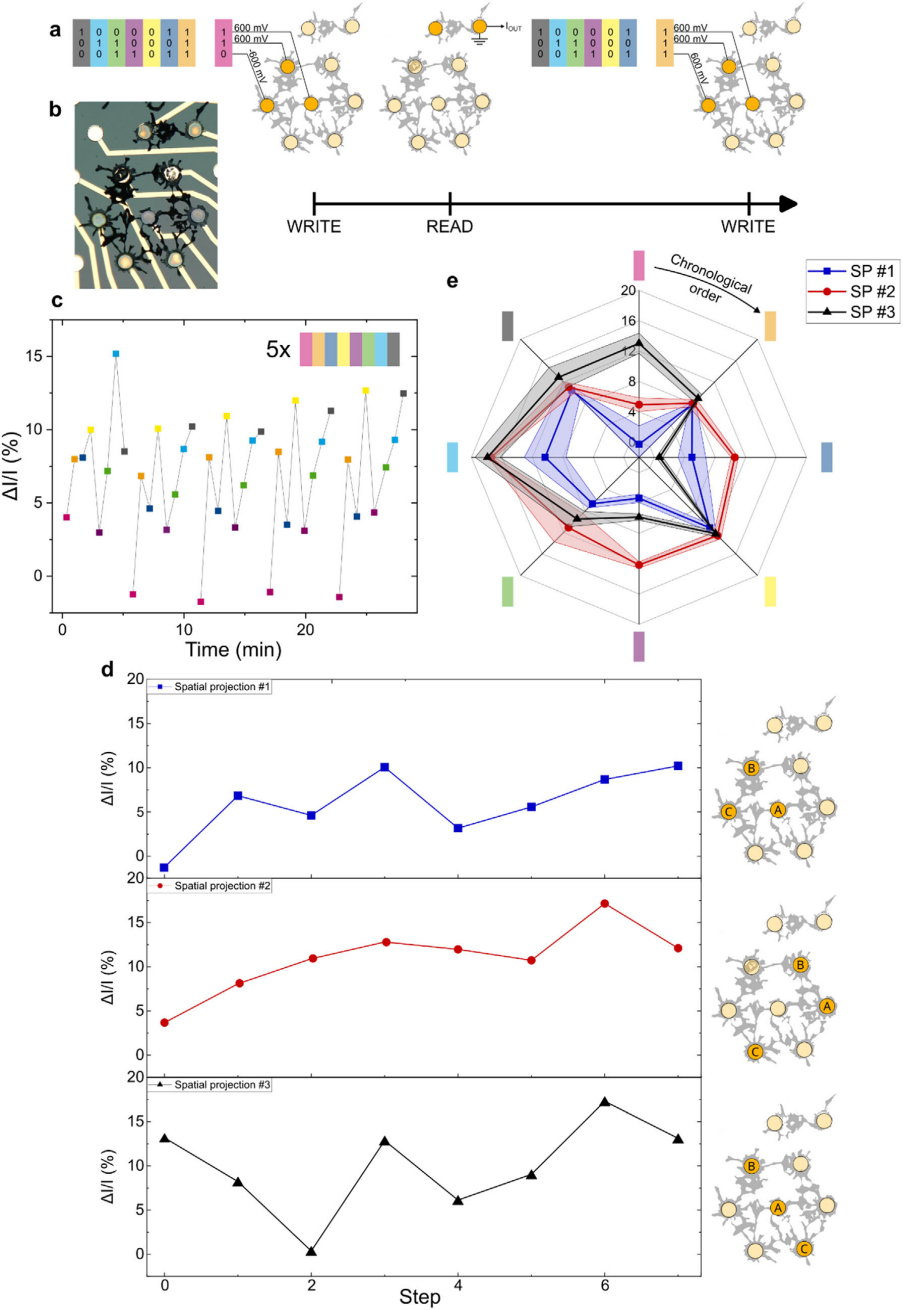
*In memory* spatial information processing. a) Schematic representation of the WRITE and READ operation sequence. Each color stands for a 3‐bit voltage pattern. b) Microscopic photo of the dendritic network used for spatial information processing. c) Output current variation for five repetitions of Spatial Projection #1. The output current variation is computed as the difference between the output current measured after a WRITE operation and the previous REST operation (all terminals set to the ground for 10 s). d) Output current variation for different sets of input electrodes receiving the temporal sequence presented in a). In the schematics of the dendritic system on the right, each letter represents the position of a single bit in the 3‐bit pattern (A,B,C). e) Spider diagram showing the average current variation and standard deviation over five cycles for three different sets of input electrodes, as presented in (d). Chronological order is shown clockwise, starting from the top of the diagram, and represented by the arrow.

First, we studied the response of a dendritic network to a single sequence of inputs, projected on different input electrodes, as introduced in **Figure** **4**. This sequence consisted of eight distinct patterns, each comprising three bits, always presented in the same chronological order (see Figure 4a). The bits were encoded as a potential versus ground, negative to represent a low bit and positive to represent a ‘1′. Each voltage pattern was applied during 10 s (“WRITE” operation) and followed by a brief “READ” operation of ≈50 ms, during which the output dendrite was biased (*V_DS_* = 100 *mV*). Between two ‘WRITE’ operations, the system was set at “rest” by grounding the three input electrodes and the two terminals of the output dendrite for 10 s (Figure 4a,b).

As the input voltage sequence was repeated, a pattern appeared in the output current of the system. Although electropolymerization is known to be stochastic, this behavior appears to be deterministic as demonstrated by cycling and conductance programming experiments (see Figures S3 and S4, respectively). If traditional figures of merit such as device‐to‐device variability do not appear to be relevant to assess the performances of dendritic systems due the uniqueness of each network, the memory abilities discussed in the following sections are still to be observed in many different devices and configurations throughout this article (an example of such behavior in two physically connected dendrites is provided in Figure S5, Supporting Information).

This cycle is best observed in the normalized current plot (Figure 4c,d), where the unique signature can be appreciated since the drift observed in the output current was removed (see Figure S6, Supporting Information for the drift). The origin of this drift was investigated, although it remains unknown (see Figures S7 and S8 and Discussion S9, Supporting Information). The modulation of current was computed as follows:

(1) $$\frac{\Delta I}{I}=\frac{I_{\mathrm{READ}}-I_{\mathrm{REST}}}{I_{\mathrm{REST}}}$$

When the same temporal sequence was projected on different input electrodes, the output of the network was modified. As this signature reflects the ability of the network to distribute ions among the various dendrites, and is thus related to the morphology of the network, this suggests that no two electropolymerized systems will give the exact same response to similar inputs, thanks to the stochasticity of the electropolymerization process.^[^ ^46^, ^47^ ^]^ The possibility to build this type of hardware on demand could have powerful implications for security purposes, for instance in the quest for physically unclonable functions.

Interestingly, even when two spatial projections were quite similar, the signature of the system in the output current can be discriminated: for spatial projections #1 and #3 (SP1 and SP3), only one of the input electrodes differed (Figure 4e), yet in both cases the output current variations were clearly distinguishable. Figure 4d indeed shows that each spatial projection signature was different and reproducible over five cycles.

In addition, this variation depends on the position of the input electrodes. The maximum increase in current was reached at step 3 for SP1 (≈10%), whereas it happened at step 6 for SP2 and SP3 and was more consequent (slightly superior to 15%). In other words, the same voltage pattern did not have the same effect on the output of the system depending on which input electrodes were addressed. This means that, given only the output current on one electrode, the system would be able to discriminate between different sources of information. This amounts to a wireless electrochemical communication system, where information can be transmitted through the electrolyte, and each source of information could be identified by its unique signature. Such spatial projection of the input patterns also demonstrates that nonlinear functions emerge when the topology becomes more complex. In comparison to the linear MAC operation performed with the Y‐shaped device, multi‐terminal devices cannot be accurately described as a linear combination of the inputs. In fact, the (1,1,1) and (0,0,0) input patterns did not result in the maximum and minimum modulations of current, as would be expected from a linear device.

#### 2.4.2 Temporal Information Processing

**Figure 5:**
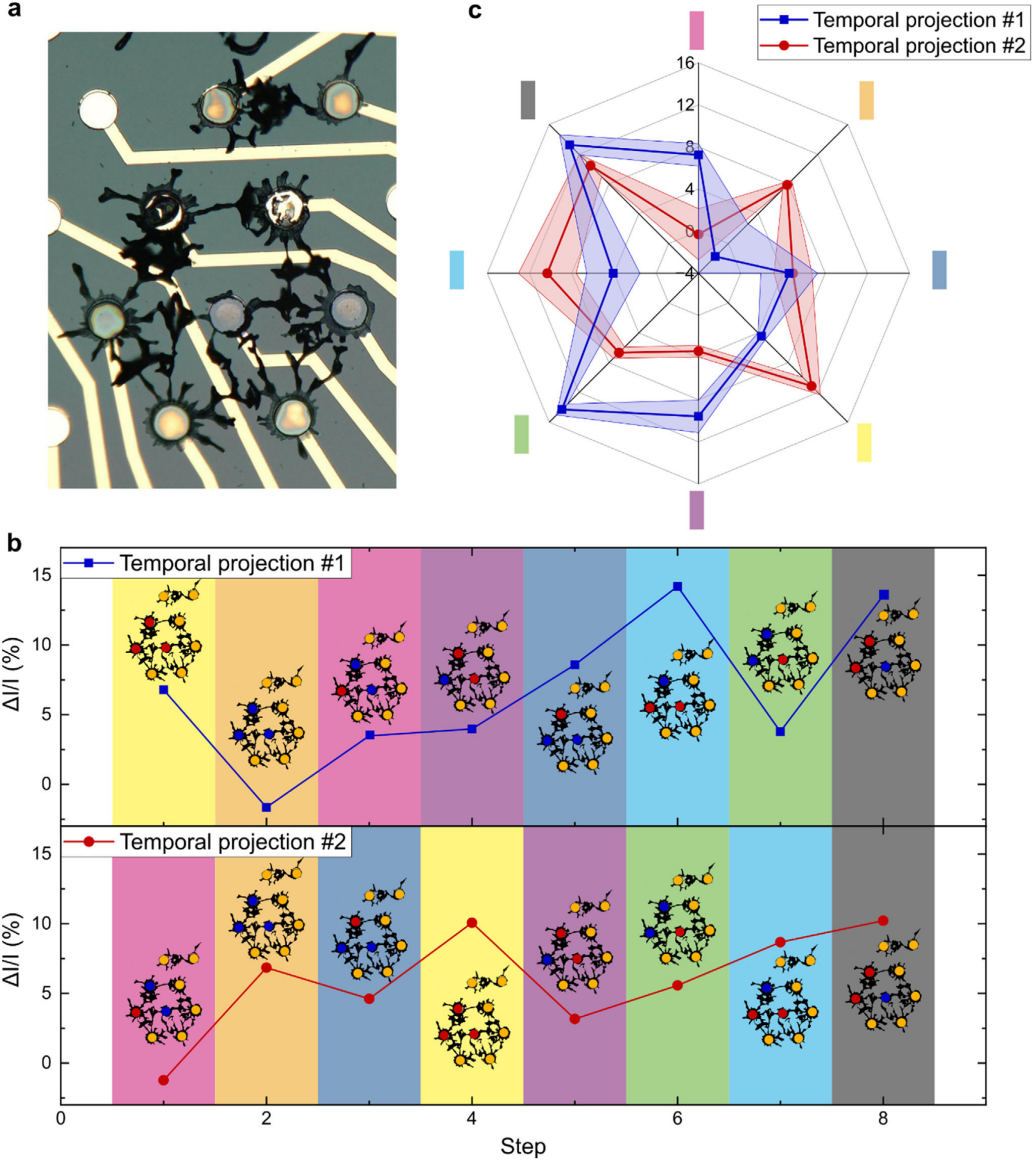
*In memory* temporal information processing. a) Microscopic photo of the dendritic network used for temporal information processing, the same as in Figure 4. b) Output current variation for different temporal sequences (the 3‐bit patterns are the same, only shuffled) presented to the same input electrodes in the system. Each color represents a 3‐bit pattern. Inset: schematic representation of the dendritic system, for which blue represents a HIGH logic state (physically encoded as 600 mV), and red represents a LOW logic state (physically encoded as −600 mV). c) Spider diagram showing the average current variation and standard deviation over five cycles for two different temporal sequences. Note that following the diagram clockwise does represent the chronological order for temporal projection #2, but not for #1.

If spatial information discrimination allows for the identification of the emitting source, dendritic networks are also able to encode temporal information. Indeed, **Figure** **5** illustrates that, even if two sequences are sent to the same input nodes, the chronological order in which patterns are presented to the system may influence the output. Using the same structure as for spatial information processing (Figure 5a), we demonstrate that the output of the system is state‐dependent.

Figure 5b shows that a single voltage pattern can have very different contributions to the output of the system based on the history of the inputs that have previously been presented to the structure. For example, for both temporal projections, the second voltage pattern presented to the dendritic network was identical (the (1,1,1) pattern represented by the orange color). In the first case, the system appeared to have been potentiated by the first pattern of the sequence. Therefore, the second voltage pattern had a negative contribution to the output current. However, for the second temporal projection, the output dendrite had already been depressed by the first voltage pattern of the sequence. As a consequence, the modulation of current following the second pattern of the sequence (the orange one) was in that case positive.

Each voltage pattern thereby had a different influence on the output current variation based on the internal state of the system. Figure 5c shows that, in order to be interpreted correctly, the whole input sequence needs to be captured. Indeed, reading the output related to only one voltage pattern will not allow to identify the corresponding input voltage pattern, as its signature may vary depending on the internal electrochemical doping state of the output dendrite, and thus on the previous voltage events that took place within the system. If the network happened to be memoryless, the output current variation would not depend on the chronological order of the input sequence, as the variation of current is always calculated compared to the previous resting state, as explained in the previous section.

This behavior of dendritic networks could have a tremendous impact on information processing: by only reading the output current of a single dendrite, within a characterized dendritic network, a dendritic device would be able to provide both spatial and temporal information. This is due to the highly complex and interwoven interactions that arise within such networks sharing a common electrolyte, a communication medium that allows the exchange of information between physically disconnected parts of the network.

### 2.5 Hardware‐Dependent Functions

Exploiting the intrinsic randomness of a manufacturing process to generate a unique output considered to be a signature of a device is an idea that has important repercussions for security purposes, and that has already been explored to implement Physical Unclonable Functions.^[^ ^35^, ^48^ ^]^ In that regard, electropolymerized networks have plenty to offer, given the hardware‐dependent functions that emerge as a consequence of the particular distribution and morphology of the polymer fibers within the network. The patterns emerging from the spatial and temporal projections in the previous section of this article could indeed be considered as a signature, a unique identification key: for the desired output to be generated, the correct input must be provided to the network, both in terms of the combination of input electrodes and the temporal sequence. Moreover, as electropolymerization is an easy‐to‐perform process, this means that the signature of the device can be generated by the end‐user itself, making him the only one in possession of the unique signature of the device.

**Figure 6:**
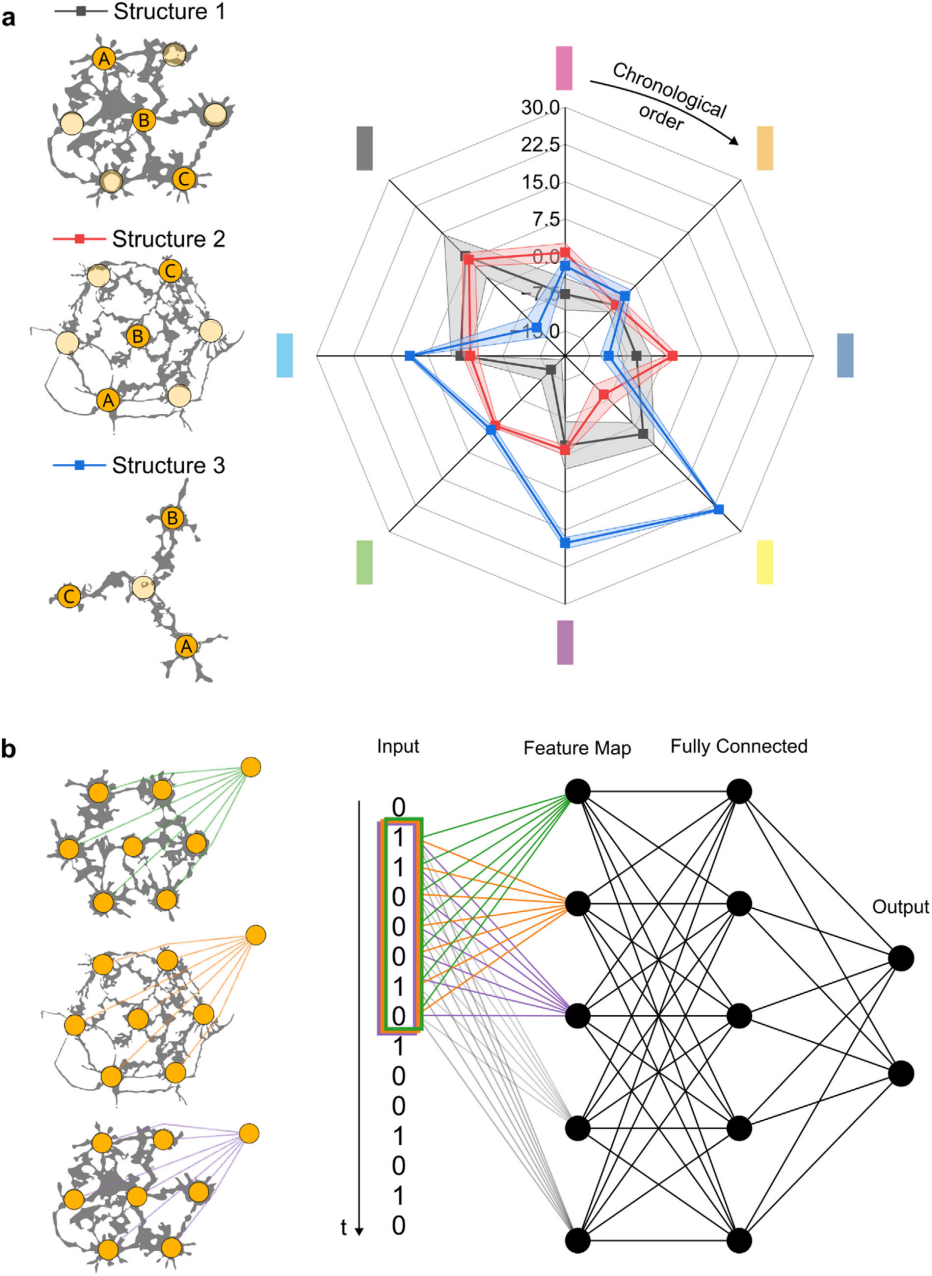
Dendritic networks for AI applications. a) Different polymer dendrite networks exhibit different behaviors. Structure 1 and Structure 2 present similar topologies but with different fiber morphology (the two structures were grown at 80 and 500 Hz, respectively). Structure 3 presents a Y‐shaped network. The spider diagram shows the average current modulation and standard deviation of each system to the same input sequence (see Figure 4 for the sequence) over 10 cycles for Structure 1 and 2, and over 5 cycles for Structure 3. Chronological order is shown clockwise, starting from the top of the diagram, and represented by the arrow. b) Each dendritic structure can be used to implement the filtering layer of a traditional AI architecture, effectively realizing a convolution operation. Each network therefore acts a kernel with a dimensionality equal to the number of input electrodes in the system. These dendritic filters feed into a conventional fully connected neural network with n input neurons, where n is the number of dendritic structures used for filtering.

In this section, we demonstrate that each dendritic network exhibits distinct functional properties and acts as a unique analog filter, as exposed in **Figure** **6**. Figure 6a demonstrates that different dendritic structures, although they have similar topologies, still behave differently. Two topologically‐similar networks were grown on each side of a unique dendrite that was used as the readout of both systems, one with thick dendrites (growth frequency of 80 Hz) and the other with thinner objects (growth frequency of 500 Hz). A third Y‐shaped structure was also used for comparison.

The same sequence of patterns as presented in Figure 4 was applied to all three networks. This resulted in different outputs, based on which network was addressed, as shown in Figure 6a. While some of the pattern signatures were very similar for the two comparable structures (notably the orange, purple, light blue and grey ones), the behavior was divergent for the other inputs. Indeed, the green and pink patterns triggered a positive modulation of the output current for Structure 2, while in Structure 1 they had a negative contribution. Consequently, over the whole sequence, each network possesses its own unique signature. The two networks, although very similar, still can be differentiated by their electrical behavior even when presented identical inputs. Due to its distinctive topology, the signature of Structure 3 strongly differs from that of the first two networks. Thanks to this property, a single readout device could thus be used to read a whole constellation of dendritic networks sharing the same medium, as each network would have a unique input‐output relationship with the readout.

Two important conclusions can be drawn. First, although the morphology of the fibers themselves is impossible to control finely, as it has been mentioned elsewhere,^[^ ^26^ ^]^ it is still possible to define the topology of the network from a global standpoint. Indeed, the connections between the nodes have essentially been maintained: although the conductance of a specific dendrite might be harder to define, two nodes can be connected from a binary perspective. Second, two different networks, however similar they might look, are not identical. If this holds from a physical point of view, this is even clearer when comparing their electric behavior. This ensures the uniqueness of each network grown on such a platform as the one presented in this article, which is at the same time a bane for applications such as deterministic logical functions and a boon (e.g., for cryptographic functionalities or classification problems). Indeed, each dendritic system can thus act as a unique transfer function through which information can be projected into a high dimensional space thanks to the nonlinearities of the networks. The nonlinear transformation carried out by the material itself could be taken advantage of to implement filters as the first layer of a neural network architecture, as illustrated in Figure 6b. Given the uniqueness of each dendritic system, ensured by the stochastic nature of electropolymerization, and the structure‐property relationships demonstrated in this class of material, each filter should present a unique response.

Each dendritic module provides a different projection of the signal depending on its topology. There is evidence that topologies are deeply rooted in computing functions such as in^[^ ^49^ ^]^ and,^[^ ^45^ ^]^ and more generally through evolution theory, which suggests that complex computing functions emerge from specialized topologies of simple building blocks. In the framework of neural networks, this link between topologies and computing performances (i.e., finding the optimal topology, or the lottery ticket theory) is still a grand challenge that bottom‐up approaches such as the one described in this paper could contribute to address. Notably, finding computing primitives that lead to complex topologies could be experimentally addressed with this approach.

A set of small dendritic systems, such as those presented in this manuscript, could therefore be used to implement feature extraction from input data, reminiscent of a 1D convolution layer in conventional neural networks.^[^ ^50^ ^]^ Using a temporal sequence encoded in the form of voltage pulses as the input, each filter projects the information into an n‐dimensional space, where n is the number of filters used to process the signal. The feature map layer can then be passed through a conventional feed‐forward architecture with learnable synaptic weights. Such architecture would leverage both the energy‐efficiency of wetware to implement physical kernels as well as the computational power of conventional hardware. In this way, the wetware presented in this manuscript becomes compatible with existing technologies, and computationally powerful concepts such as backpropagation can still be implemented effectively. Nevertheless, and as for now, the lack of a theoretical foundation hinders the scale up of this technology. Although some work can be found discussing growth modeling,^[^ ^46^, ^51^ ^]^ conductive polymer dendrites remain mostly studied from an experimental perspective.^[^ ^26^, ^32^, ^33^, ^47^, ^52^, ^53^ ^]^ Interesting studies have brought to light the structure of thin‐film PEDOT:PSS or the charge‐compensating interactions between ionic and electronic charge carriers,^[^ ^54^, ^55^, ^56^, ^57^, ^58^, ^59^ ^]^ but very often these studies necessitate access to specific equipment and might be prevented by the unconventional 3D geometry of polymer dendrites (for instance, Figure S10, Supporting Information shows the Raman spectra of a PEDOT:PSS fiber recorded at two different spots).

## 3 Discussion

Biological neural networks are an important source of inspiration when it comes to efficient computing, mainly due to their innate ability to massively parallelize information processing. If large artificial neural network models have proven to be very efficient computationally, they are still far from the energy efficiency of biological brains. In these models, performance comes with high accuracy in the synaptic weights and dense interconnections between layers of neurons that translate into important energy consumption. In biological networks, only useful connections are grown and result in sparse and specialized topologies that optimize resource use. Additionally, these topologies possibly bear more profound memory representation that connectionist models based solely on synaptic weight evolution during learning cannot capture. Topologies set the dimensional space in which synaptic plasticity can be expressed, and could possibly be the foundation for semantic representations (i.e., higher level of organization in the representation of concepts).^[^ ^60^ ^]^ Our work highlights the role of topologies in the spatiotemporal projection of information and could be a framework to reconciliate connectionist and semantic approaches. We show in this paper that the control operated over the topology of dendritic networks governs the complexity of the operation of the system, due to the interplay between form and function. In other words, if small networks implement nearly linear functions, such as the MAC operation discussed earlier, more developed systems exhibit nonlinear behaviors that are more complex to implement but offer greater computational power. The bottom‐up approach of dendritic growth therefore allows the function of a multiterminal device to be defined by its physical structure, as complexity emerges from the physical structure of the network itself.

Another concept seldom discussed in the computing community is the wide diversity of synapses and information carriers in the brain. Although a great number of neuromodulators has already been identified,^[^ ^61^ ^]^ the underlying mechanisms by which they participate in information processing remains unclear. Especially striking is the varied nature of these, from peptides to gaseous molecules. Apart from neurotransmitters, information also appears to be conveyed by marker molecules, as it has been proposed that their gradient of concentration could guide axonal growth in the early stage of brain development.^[^ ^62^ ^]^ This sharply contrasts with the current view in computing, where electrons are the only information carrier. Using an electrochemical platform such as the one presented in this article, the number of information carriers in the system could be increased drastically, as many biomolecules have already been demonstrated to modulate the behavior of organic electrochemical transistors.^[^ ^63^, ^64^, ^65^, ^66^, ^67^ ^]^ With recent advances regarding degradability and disintegration of organic polymers,^[^ ^68^, ^69^, ^70^ ^]^ full structural plasticity could also become a way to store information directly in the structure of the network, making the most out of the limited resources of the system by destroying unnecessary synapses to reemploy the monomer molecules more efficiently. Although long term depression could be emulated by overoxidizing polymer fibers,^[^ ^15^ ^]^ full structural plasticity in dendritic networks is yet to be implemented (see Figure S11, Supporting Information). This could further improve the computational power of such systems, since bottom‐up approaches appear to be very promising at solving complex optimization problems, as it was demonstrated with multi‐agent evolutionary networks.^[^ ^49^, ^71^ ^]^

The ability to learn and evolve is an incredibly potent property of the brain, one that the current top‐down approach of solid‐state electronics cannot emulate. Evolving systems have the great advantage to adapt to the tasks at hand, so that resources are employed as efficiently as possible. In such systems, complex behavior arises from simple local rules, as it was proposed with cellular automata.^[^ ^72^ ^]^ Using the computational power of adaptive bottom‐up physical systems has been explored with NanoCells^[^ ^73^, ^74^, ^75^ ^]^ or slime mold,^[^ ^4^, ^76^, ^77^, ^78^, ^79^ ^]^ but the physical structures in these examples were obtained without any physical relationship with the computation to be performed. Here, the versatility of electropolymerization offers to adjust the operation of the device as needed *in operando*,^[^ ^26^ ^]^ enhancing its capabilities and bringing organic electronics one step closer to biology by implementing local learning rules such as Hebbian principles.^[^ ^45^ ^]^ Implementing this technology in a solid‐state electrolyte could make solid‐state electronics more plastic and create new possibilities with the integration of a bottom‐up additive approach to otherwise deterministic electronics. Indeed, the stochastic growth and process variability of organic electronics, which are inconveniences in the realm of traditional electronics and digital computing, turn into opportunities when it comes to unconventional computing. Having systems which growth is not fully controllable, and which behavior cannot be anticipated is highly valued for cryptographic and security applications such as Physical Unclonable Functions (PUFs), which take advantage of variability in production to generate devices that present a unique signature.^[^ ^35^ ^]^ Although still a recent concept, conductive polymer dendrites show great potential for the physical implementation of unconventional computing and the integration with solid‐state electronics. However, we anticipate that scaling up this technology will face three major challenges. First, a better understanding of conducting polymer dendrite growth will be needed to address concerns regarding controllability. Previous studies have already demonstrated the influence of electrical parameters,^[^ ^26^ ^]^ and future work will consider the influence of the electrochemical environment over the morphology and properties of such objects, as this knowledge should enhance reproducibility. Second, integration efforts will require downsizing the structures (see Figure S12, Supporting Information). Apart from limiting their footprint, downsizing the dendrites should enhance computational power by increasing the complexity of the networks and operational speed as the capacitance of these objects will scale with their volume. Lastly, from a computational perspective, electropolymerization opens up the door for the development of a new class of evolutionary devices. As a versatile fabrication tool, it allows the implementation of a bottom‐up fabrication strategy (Figure S13, Supporting Information shows different topologies implemented on various MEAs). In that regard, electropolymerization offers a level of control and flexibility that standard top‐down techniques cannot reproduce. In addition, these structures have the potential to evolve in operando, further enhancing their computing abilities.^[^ ^80^ ^]^ The freedom offered by such evolutionary systems comes at a cost: growing dendritic networks on multielectrode arrays means that the fibers are exposed to an open‐space wet environment and to antenna effects, contrary to thin‐film technologies that are encapsulated between two electrodes. Overall, energy consumption could benefit from the downscaling of dendritic systems (see Figure S12, Supporting Information), although it should be noted that an aqueous electrolyte remains an intrinsically noisy environment that could limit downsizing. According to estimations made with similar materials, the switching energy (i.e., energy required to inject or repeal ions into or from the electrolyte to induce a change of conductance in the PEDOT‐based material) is expected to reach a few tens of attojoules,^[^ ^81^ ^]^ outperforming memristive crossbar arrays,^[^ ^82^ ^]^ and matching the performance of CMOS‐ring oscillators and spin‐torque oscillators,^[^ ^83^ ^]^ as well as getting close to state‐of‐the‐art neuromorphic photonic systems.^[^ ^84^ ^]^ Regarding energy consumption, the cost of producing the devices is very often discarded. Yet, the electropolymerization framework allows to get rid of energy‐intensive cleanroom equipment, as it is compatible with usual benchtop waveform generators.

## 4 Conclusion

Our work establishes electropolymerized PEDOT:PSS dendritic networks as a new class of morphology‐dependent hardware, which properties not only arise from the material but also from the morphology of the fibers and the topology of the network. We highlight the complex interplay between form and function in these structures.

We bring to light that these objects present a nonlinear and asymmetric electric behavior directly related to their morphology and the ionic distribution across the system, which depends on the activity of all the fibers of the network. PEDOT:PSS dendritic networks also appear to possess an intrinsic memory that, coupled with the self‐gating and inter‐gating effects of polymer dendrites, allows the system to perform *in materio* computing tasks, such as spatiotemporal information processing. Indeed, conductive polymer networks are able to discriminate the source of surrounding voltage events and present state‐dependent computing abilities, as in such systems the output of the network seems to be conditioned by past inputs. Finally, we evidence that these properties, coupled with the stochasticity of AC‐electropolymerization, allow the growth of networks with similar large‐scale topologies, but in which the distinct morphology of each dendrite gives rise to a unique granularity, and consequently to a distinctive electric behavior. However, this technology is still under development, and it appears essential to establish the range of variability within which such structures can operate reliably, thereby enabling probabilistic hardware capable of consistently and reproducibly supporting computing applications.

Electropolymerization definitely emerges as a fabrication method of choice in the quest for a new paradigm that could help solve the hardware crisis at hand, as it offers the possibility to create complex structures with minimal resource requirements, implementing an effective bottom‐up approach. With close to no need for complex and costly equipment and offering incredible simplicity, it holds the potential to foster a new era of organic, cost‐effective and bioinspired electronics.

## 5 Experimental Section

### Substrate Fabrication

The MEAs used as a substrate for the electropolymerization of dendritic networks were fabricated as described elsewhere.^[^ ^28^, ^29^ ^]^

### Dendritic Growth

Dendritic growth was carried out through AC‐electropolymerization using a Keysight 33600A Trueform waveform generator. Square waves with a frequency of 80 Hz and a peak voltage amplitude of 5 Vp were the default waveform applied between the working and grounded electrodes, unless otherwise specified. For the growth to happen, the system was immersed in an aqueous solution containing 1 mM of poly(sodium‐4‐styrene sulfonate) (NaPSS), 10 mM of 3,4‐ethylenedioxythiophene (EDOT), and 10 mM of 1,4‐benzoquinone (BQ). All chemicals were bought from Sigma–Aldrich and used without further modification. The growth of the system was monitored though a TrueChrome Metrics camera.

### Electrical Characterization

All the electrical characterizations presented in this work were conducted on an Agilent B1500A Semiconductor Analyzer coupled with a B2201A Switching Matrix.

### Microscopic Images

All microscopic pictures were taken using a Keyence VHX‐6000 microscope.

## Conflict of Interest

The authors declare no conflict of interest.

## Author Contributions

Y.C., S.P., and F.A. performed Conceptualization. C.S., Y.C., S.P., and F.A. performed Methodology. C.S. performed Investigation. C.S. performed Visualization. Y.C., S.P., and F.A. performed Supervision. C.S. wrote the original draft. C.S., Y.C., S.P., and F.A. wrote, review and edited the final manuscript.

## Supporting information



Supporting Information

## Data Availability

The data that support the findings of this study are available from the corresponding author upon reasonable request.
