# Supplementary material for: Brain‐Inspired Polymer Dendrite Networks for Morphology‐Dependent Computing Hardware
Source: Adv Sci (Weinh). 2025 Aug 11;12(33):e02291. doi: 10.1002/advs.202502291 (PMC12412553; doi:10.1002/advs.202502291)
Supplement: Supplementary file 1 — Supporting Information [file ADVS-12-e02291-s001.docx]

Supporting Information

Brain-inspired polymer dendrite networks for morphology-dependent computing hardware

*Corentin Scholaert, Yannick Coffinier, Sébastien Pecqueur and Fabien Alibart**


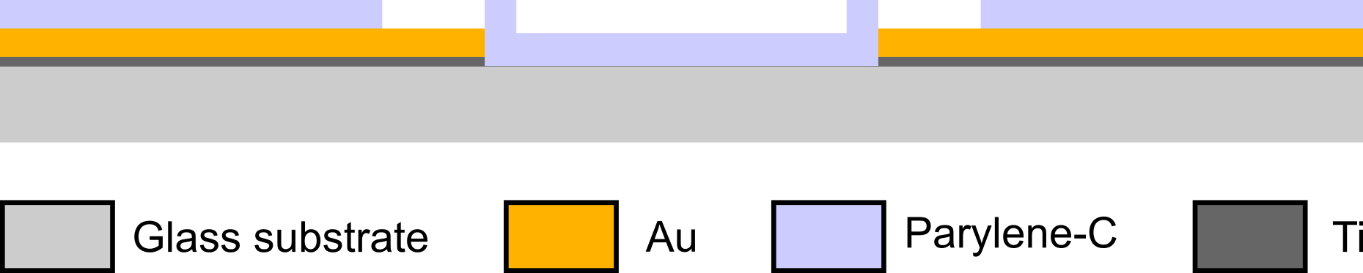


**Figure S1**. Cross-sectional diagram of a multielectrode array. A 100 nm gold layer is deposited onto a glass substrate, with a 10 nm titanium adhesion layer. A 1 µm parylene-C layer is then applied as an insulating layer. Not to scale.


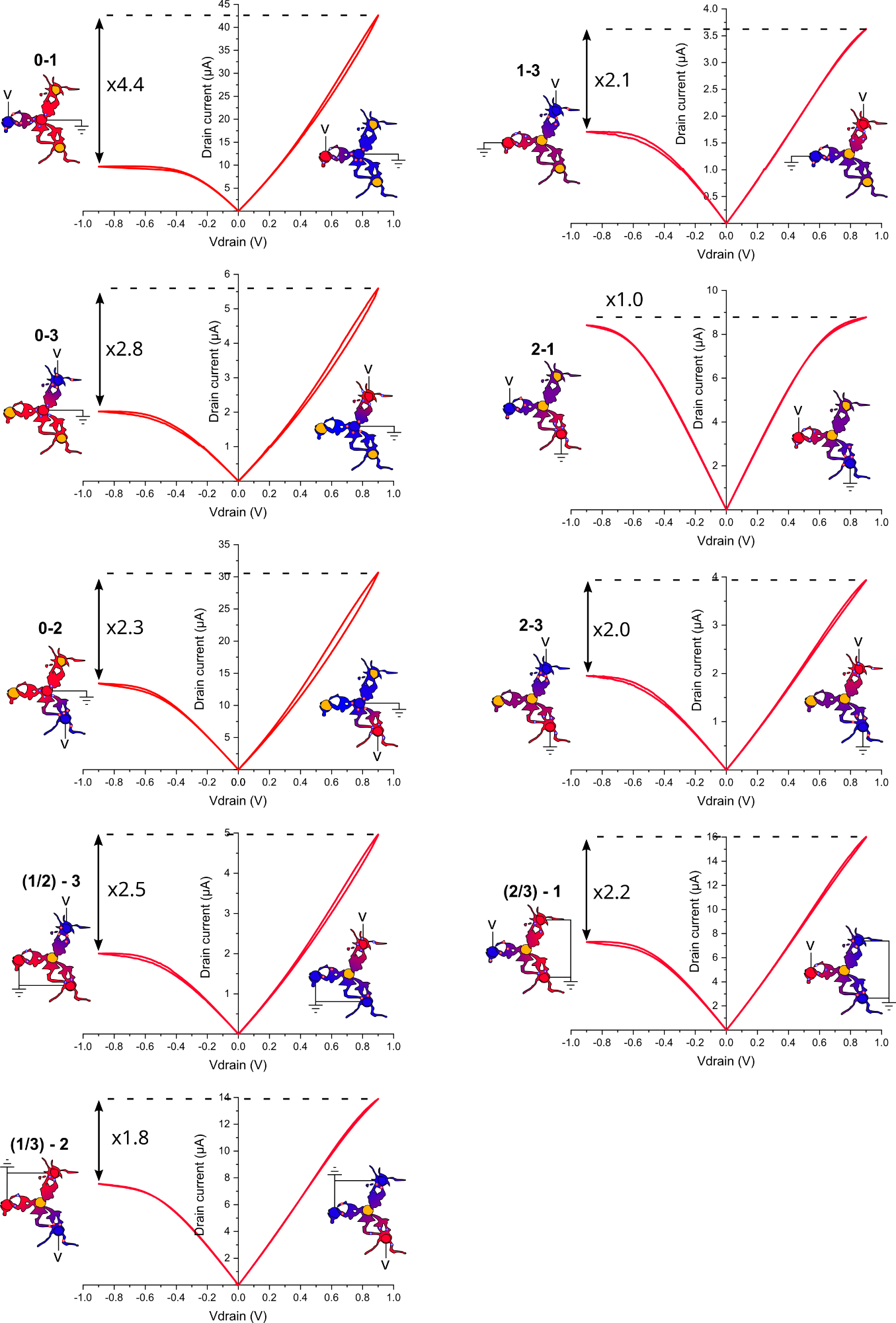


**Figure S2**. IV curves for all the different combinations of voltage distribution in a Y-shaped dendritic network. The inset figures show which electrodes are being addressed in each case.


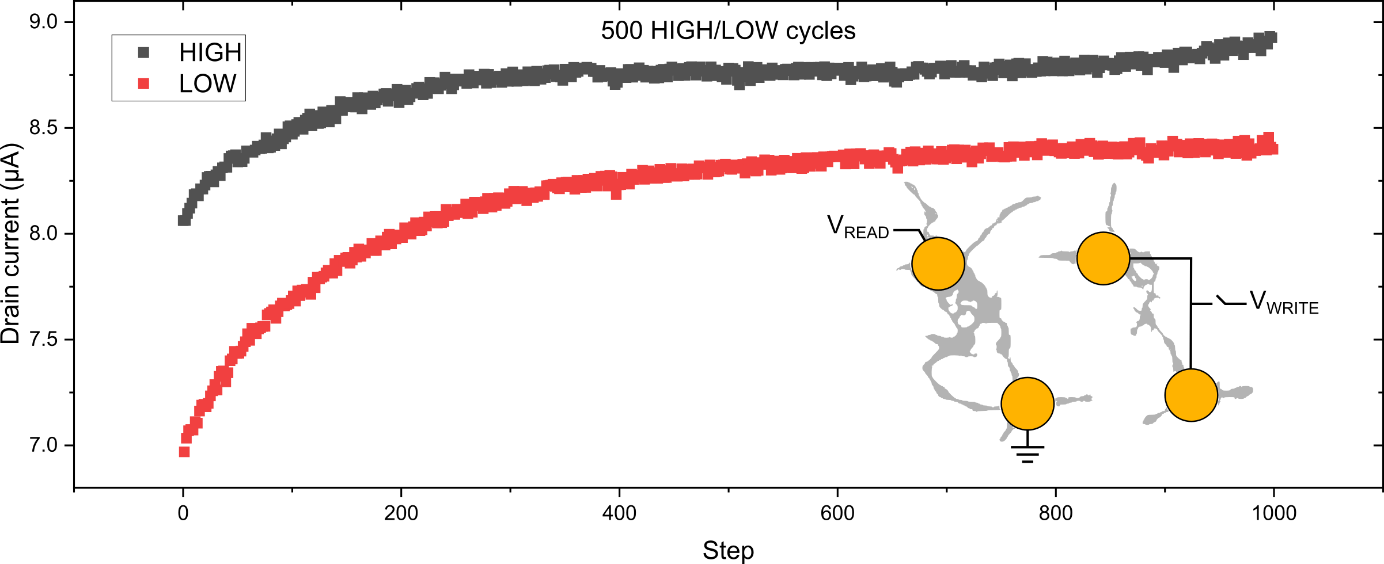


**Figure S3.** Cycling test performed on parallel dendrites. A HIGH conductance state is reached by applying V_WRITE_ = -600 mV while a LOW conductance state corresponds to V_WRITE_ = 600 mV. The write & read sequence is as described in the manuscript. N = 500 cycles**.**


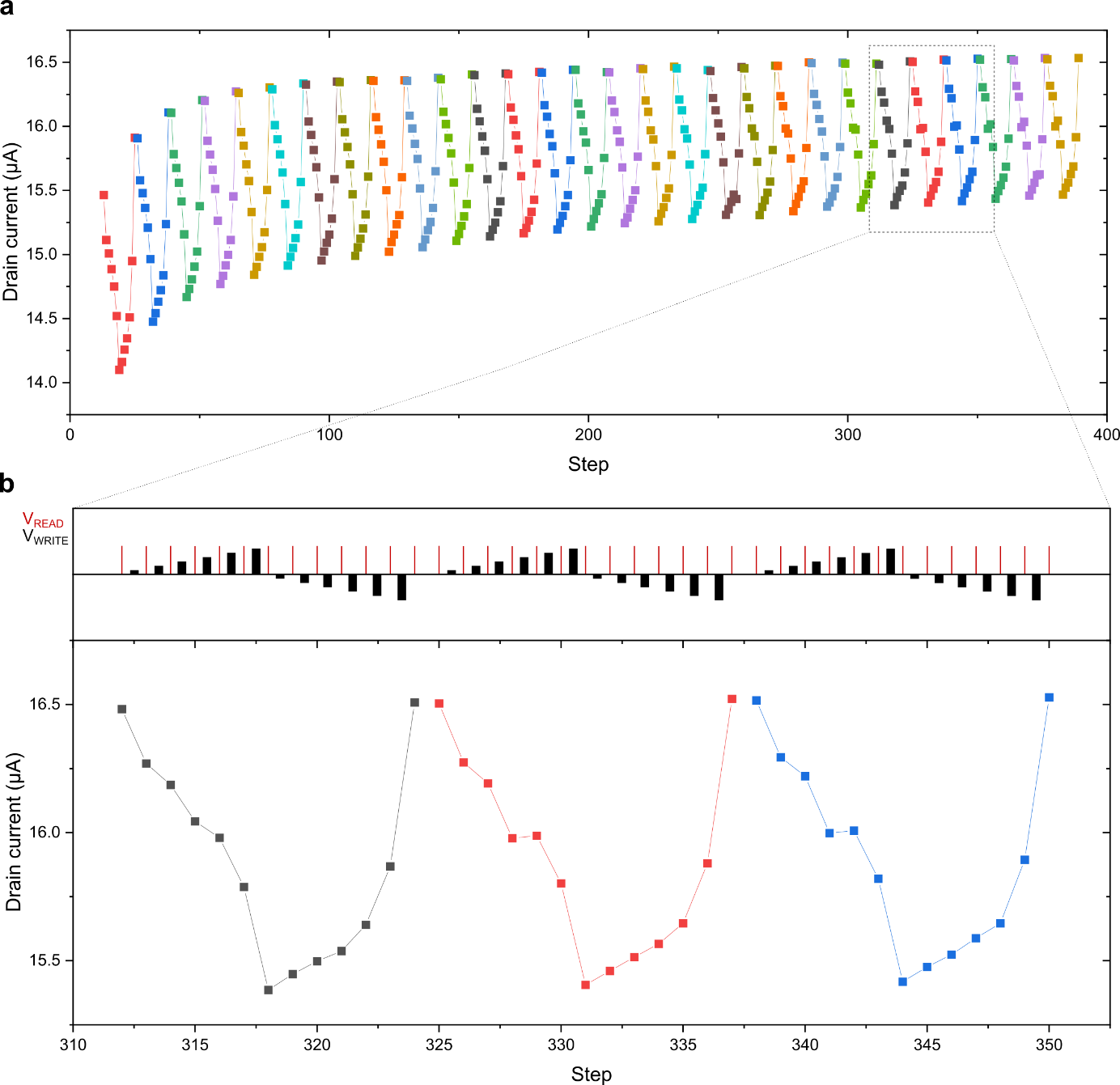
**Figure S4.** Conductance programming on two parallel dendrites. For each cycle, V_WRITE_ makes 100 mV steps, starting from 100 mV up to 600 mV and then from -100 mV to -600 mV.

**
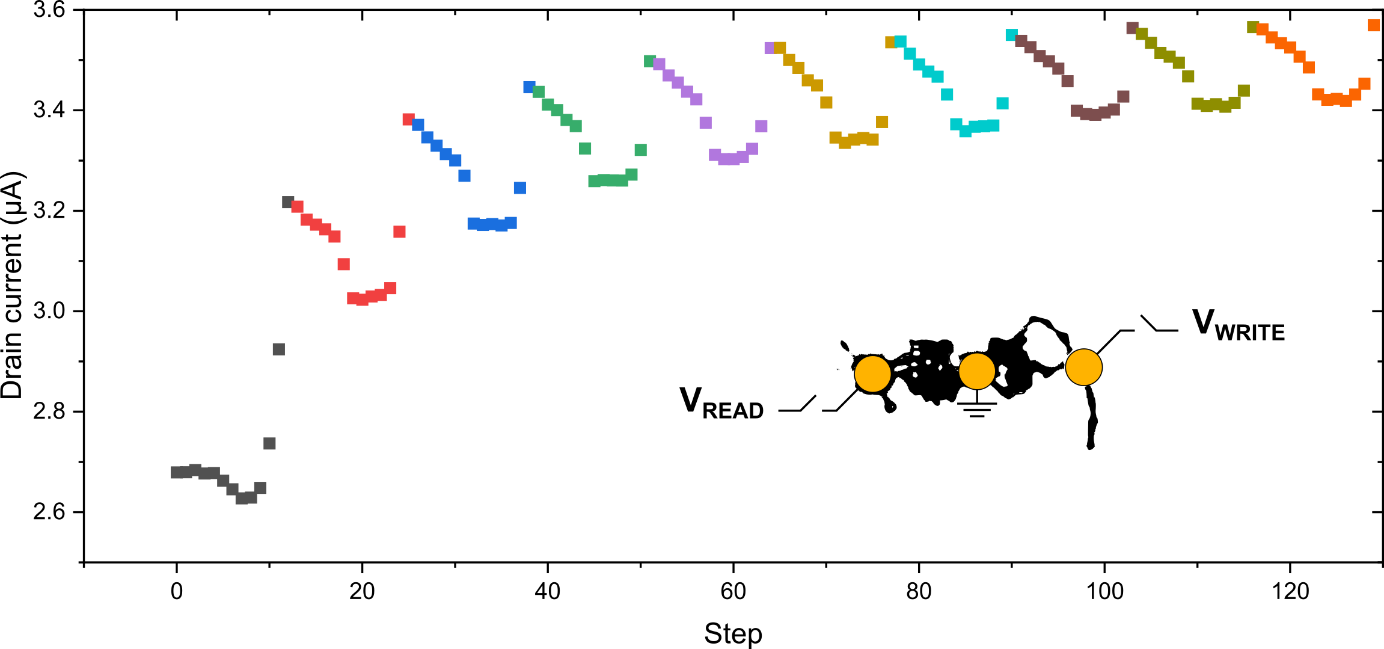
**

**Figure S5.** Conductance programming on two physically connected dendrites. A picture of the system is shown in inset. A WRITE operation is performed during 10 s, before a brief READ operation (about 50 ms). For each cycle, V_WRITE_ makes 100 mV steps, starting from 100 mV up to 600 mV and then from -100 mV to -600 mV.


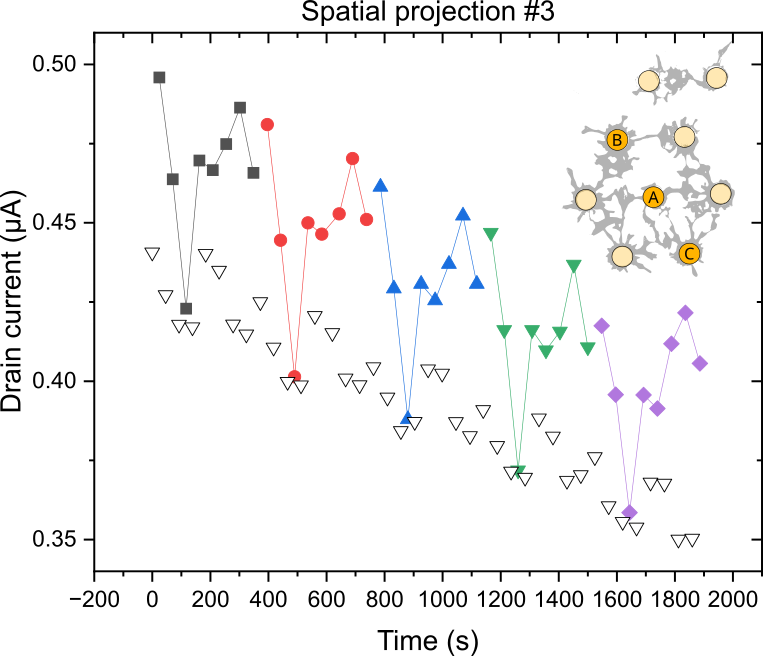


**Figure S6**. Drain current of the output dendrite of the dendritic system used for the spatiotemporal information processing. This graph brings to light the pattern that appears in the output current when an input sequence is repeated. The downward current drift discussed in the text is clearly observable. Here, each color represents a repetition of the input sequence, namely Spatial Projection #3. The white down-pointing triangles represent the output current after a ‘REST’ operation, which are used to compute the output current variations ($\Delta I/I$).


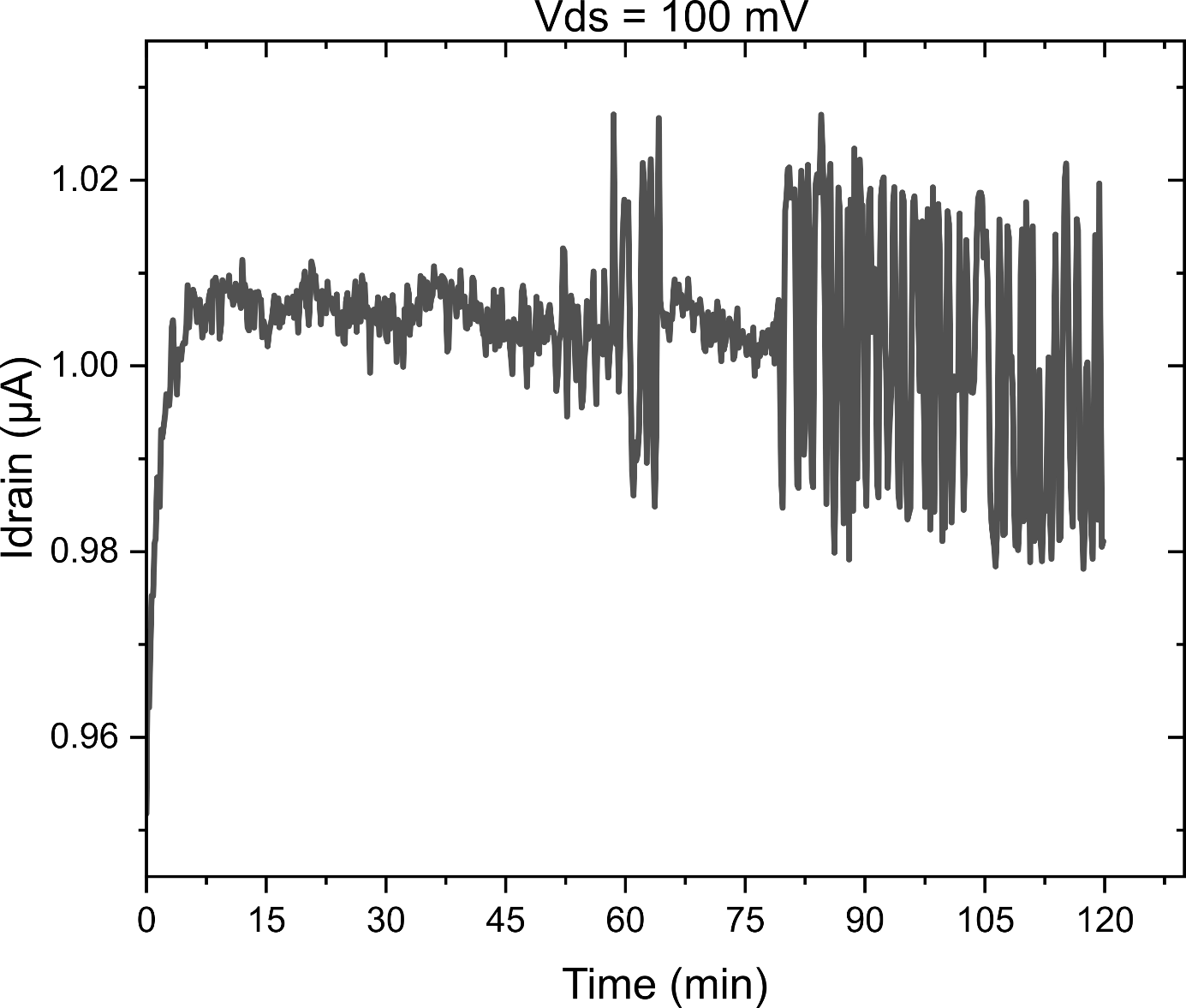


**Figure S7**. Aging test in PBS. A bias of 100 mV was applied across the terminals of the readout dendrite used in **Figure 6** for Structure 1 and 2, and the drain current was recorded. Contrary to what was observed during the *in materio* programming experiments, no consequent drift appeared during the 2h recording.


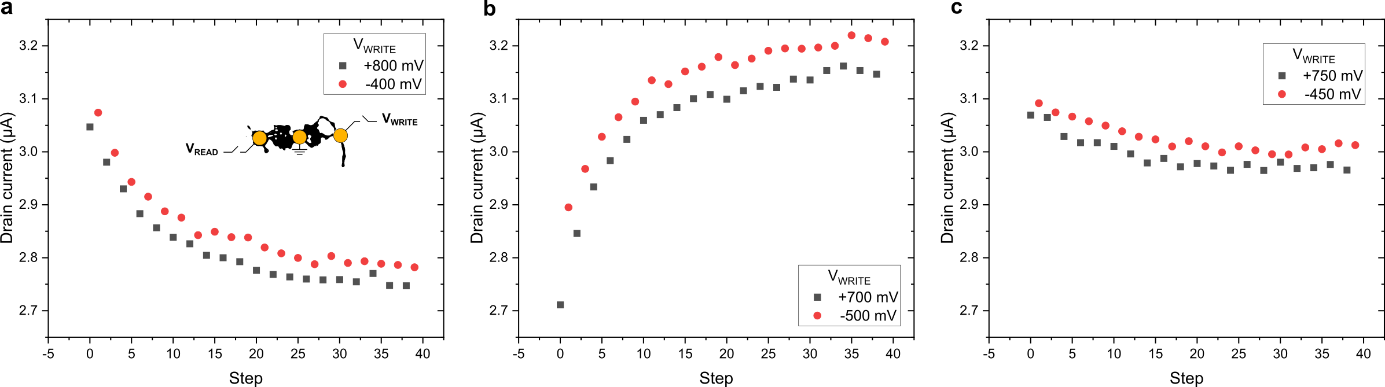


**Figure S8**. Investigation of the origin of the current drift in polymer dendrites. a) Cycling test with V_WRITE_ = 800 mV for the LOW conductance state and V_WRITE_ = -400 mV for the HIGH conductance state. Inset: representation of the experimental setup, the same as in Figure S5. b) Cycling test with V_WRITE_ = 700 mV for the LOW conductance state and V_WRITE_ = -500 mV for the HIGH conductance state. c) Cycling test with V_WRITE_ = 750 mV for the LOW conductance state and V_WRITE_ = -450 mV for the HIGH conductance state.

**Discussion S9**

As presented in **Figure S8**, two dendrites were grown between adjacent electrodes, one to be used as the input and the second one as the output. Different pairs of voltages were then tested during cycling tests to study their effect on the output current, either [-400 mV, 800 mV], [-450 mV, 750 mV] or [-500 mV, 700 mV]. In all three cases the current drift was observed and seemed to converge toward a stable value after about 20 cycles. Yet, it is interesting to note that this final value appears to depend on the chosen set of voltages. Additionally, the direction of the drift changed as the average value of V_WRITE_ decreased, from a downward direction to an upward direction (see **Figure S8a-b**). This suggests that the system could be drifting toward an equilibrium that depends on the voltages applied during the successive WRITE operations. Indeed, different dynamics are expected for the cations of the electrolyte to charge and discharge the dendrites,^[85]^ leading to different equilibriums. Interestingly, almost no drift is to be reported in **Figure S8c**.

**
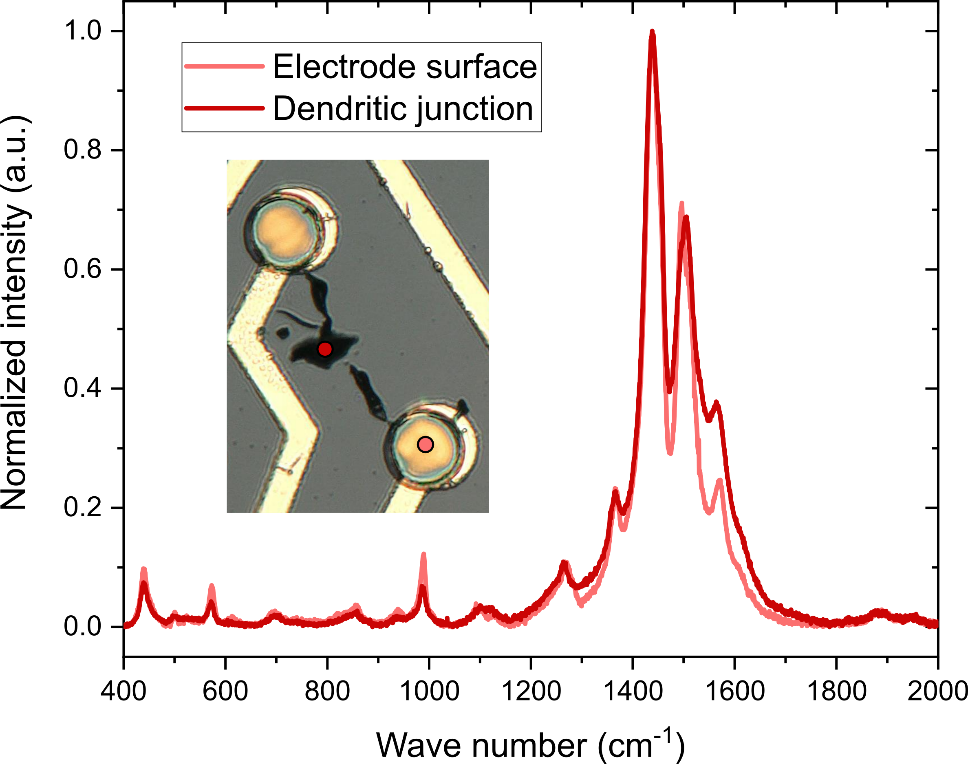
**

**Figure S10**. Raman spectroscopy on a PEDOT:PSS dendrite. Inset: picture of the dendrite, where the two dots show the positions where the spectra were recorded. As can be observed, the spectra do not perfectly overlap. It remains unclear whether these variations arise from a change in the polymer conformation depending on the position or from the cylindrical shape of the object, which may challenge the analytical capabilities of the equipment.

**
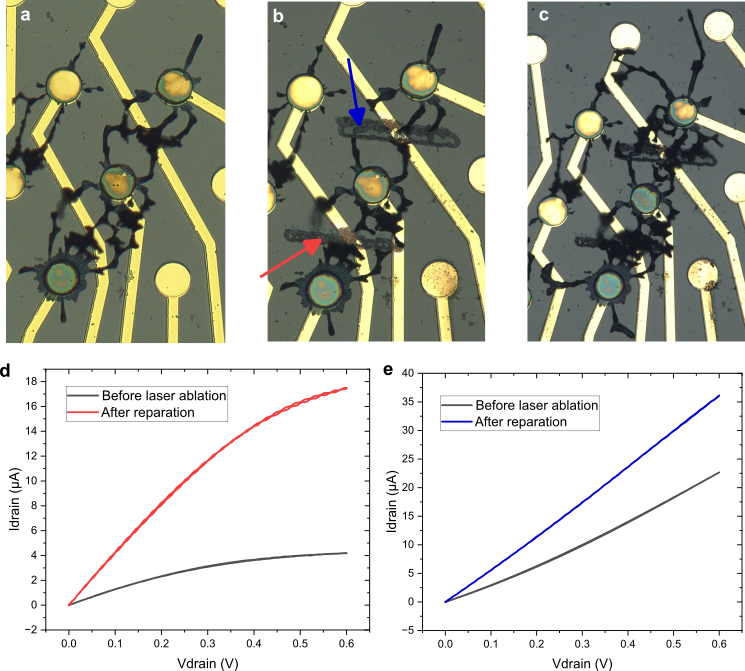
**

**Figure S11**. Full structural plasticity in polymer dendrite networks. Laser ablation was used to ensure the physical destruction of the connection. a) Polymer dendrites before laser ablation, b) after laser ablation (shown by the arrows) and c) after reparation using electropolymerization. The top dendrite is represented in blue while the bottom dendrite is represented in red.


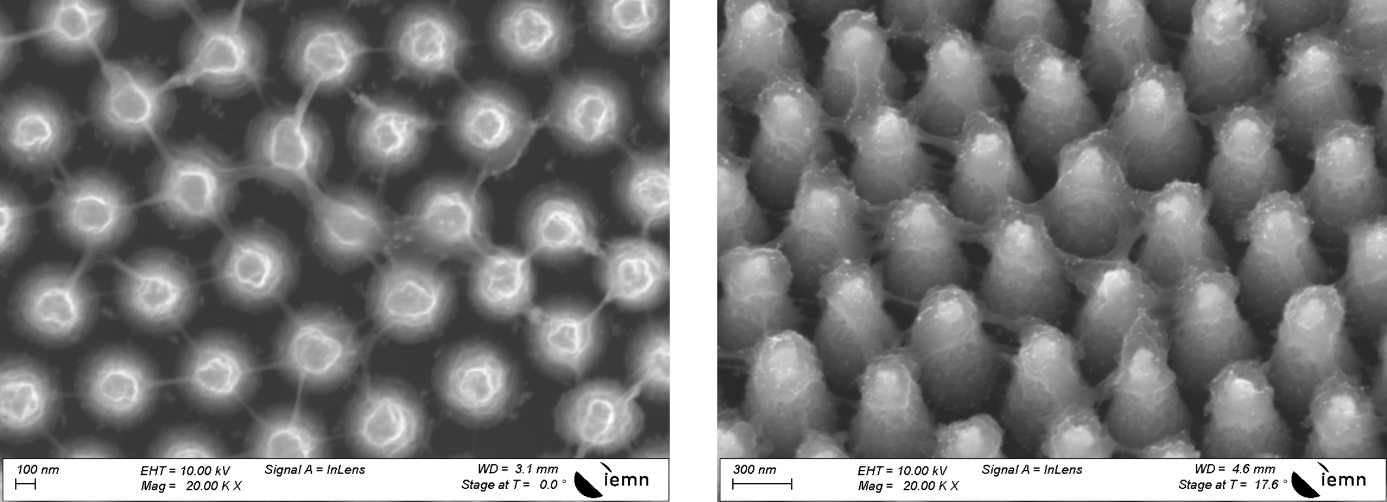


**Figure S12.** Nanoscale polymer dendrites. Nanoscale PEDOT:PSS dendrites were grown between metallized nanoneedles on a silicon substrate.


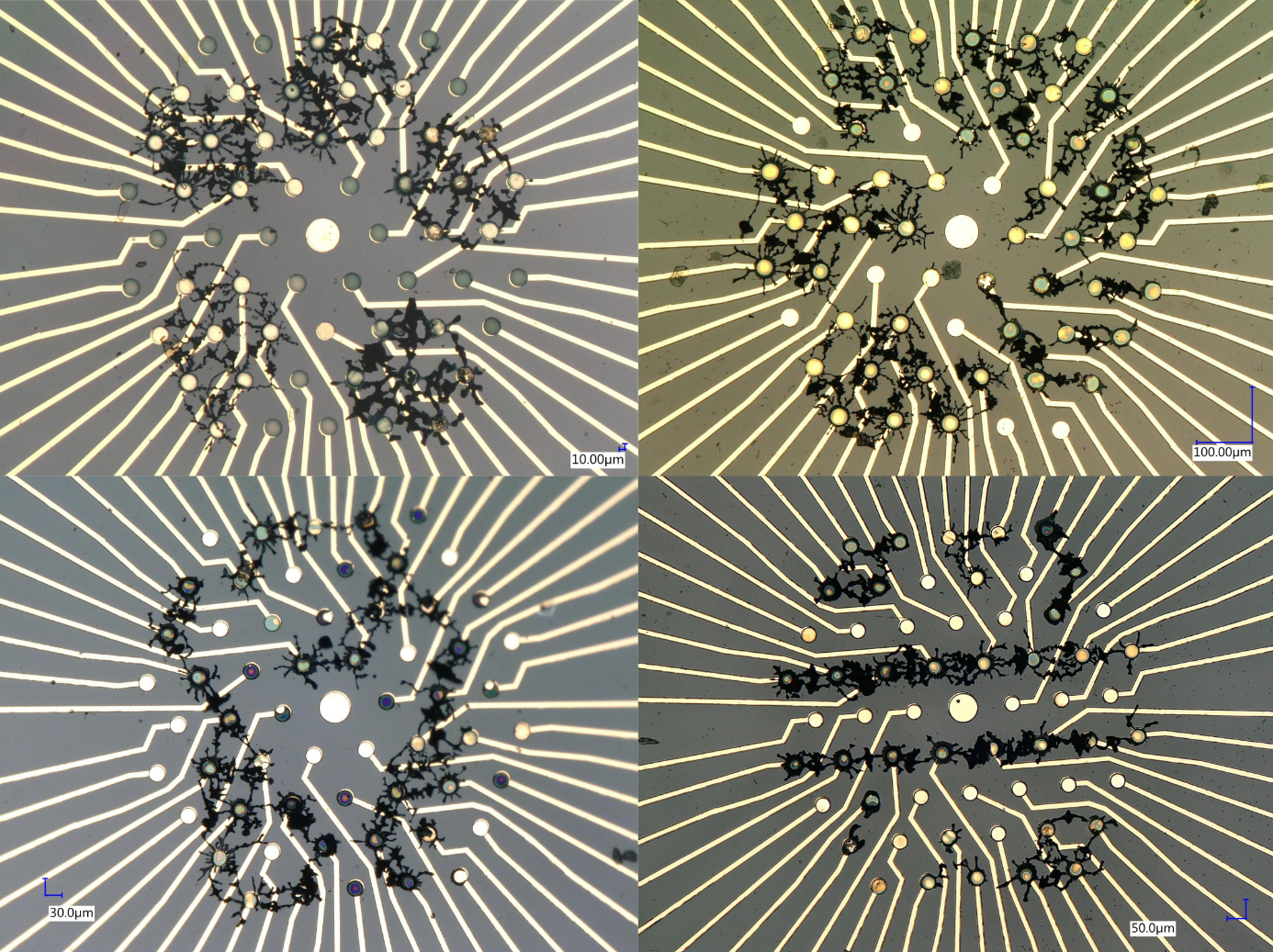


**Figure S13.** Scaling up the system. Four pictures showing different dendritic systems grown onto different multielectrode arrays, displaying the range of topologies as well as the evolving capabilities of such networks.
